# Deploying Patient-Facing Application Programming Interfaces: Thematic Analysis of Health System Experiences

**DOI:** 10.2196/16813

**Published:** 2020-04-03

**Authors:** Aaron Neinstein, Crishyashi Thao, Mark Savage, Julia Adler-Milstein

**Affiliations:** 1 Department of Medicine University of California, San Francisco San Francisco, CA United States; 2 Center for Digital Health Innovation University of California, San Francisco San Francisco, CA United States; 3 Center for Clinical Informatics and Improvement Research Department of Medicine University of California, San Francisco San Francisco, CA United States

**Keywords:** application programming interface, consumer health information, electronic health record, health information exchange, health policy, patient engagement

## Abstract

**Background:**

Health systems have recently started to activate patient-facing application programming interfaces (APIs) to facilitate patient access to health data and other interactions.

**Objective:**

This study sought to ascertain health systems’ understanding, strategies, governance, and organizational infrastructure around patient-facing APIs, as well as their business drivers and barriers, to facilitate national learning, policy, and progress toward adoption.

**Methods:**

We performed a content analysis of semistructured interviews with a convenience sample of 10 health systems known to be leading adopters of health technology, having either implemented or planning to implement patient-facing APIs.

**Results:**

Of the 10 health systems, eight had operational patient-facing APIs, with organizational strategy driven most by federal policy, the emergence of Health Records on iPhone, and feelings of ethical obligation. The two priority use cases identified were enablement of a patient’s longitudinal health record and digital interactions with the health system. The themes most frequently cited as barriers to the increased use of patient-facing APIs were security concerns, an immature app ecosystem that does not currently offer superior functionality compared with widely adopted electronic health record (EHR)–tethered portals, a lack of business drivers, EHR vendor hesitation toward data sharing, and immature technology and standards.

**Conclusions:**

Our findings reveal heterogeneity in health system understanding and approaches to the implementation and use of patient-facing APIs. Ongoing study, targeted policy interventions, and sharing of best practices appear necessary to achieve successful national implementation.

## Introduction

### Background

An array of federal policy efforts seeks to create a more patient-centered, consumer-empowered health care system by improving patients’ access to their electronic health information (EHI) [1,2]. Previous efforts to create patient-controlled health records (most famously, Google Health and Microsoft HealthVault) never gained traction, due in part to the lack of easily available, digital patient data [3]. Since then, the health care system has digitized health data significantly, with 96% of hospitals and 78% of physician offices now using certified electronic health record (EHR) technology [4]. A major national survey of consumers shows that consumers want and use electronic access to their health information, including mobile access through smartphones and use of health apps [5]. In parallel, regulations now require that patients get timely (within 48 hours) [6] access to their health records to view, download, and transmit them electronically (as opposed to only being able to receive a paper copy). Beginning January 1, 2019, with stage 3 of the federal Medicare and Medicaid EHR Incentive Programs (now known as the Promoting Interoperability Program) and bolstered by provisions of the 21st Century Cures Act, health systems must give patients electronic access to their EHI via patient-facing application programming interfaces (APIs), “without special effort” [1,7].

These patient-facing APIs have now begun to go live, enabling patients to access their EHI as well as to direct access by third-party software apps [8]. This presents a potentially pivotal moment in the evolution of the US health care system because success could break open long-siloed patient data [9], leading to better progress in interoperability, enabling patients to create their own longitudinal health record, and realizing the potential of patient engagement via an emerging ecosystem of third-party apps. However, success is not a foregone conclusion, with health systems starting to pursue patient-facing APIs amidst uncertainty and complex issues spanning policy, reimbursement, technical implementation, governance, privacy, and security [10]. As health systems wrestle with numerous decisions about how to deploy patient-facing APIs, they risk doing so in silos, without the benefit of interorganizational learning [11]. This would limit the identification and sharing of emerging best practices, as well as challenges encountered, increasing the likelihood of early failures that could deter the next wave of adopters. Capturing and sharing early experiences with patient-facing APIs is, therefore, critically important to inform ongoing policy and implementation efforts seeking to ensure that this national transition is safe and secure and has the intended impact of accelerating patient data access, improving quality of care, and increasing options for tools available to digitally engaged patients.

### Objectives

We sought to identify and summarize how early adopter health systems are planning for and implementing patient-facing APIs. We specifically wanted to ascertain their understanding and their overall strategies and key use cases, including the associated organizational infrastructure, governance, policies, and processes, and how their approaches are shaped by policies and other external drivers. Finally, we aimed to identify barriers encountered in these early experiences that could inform policy and practice efforts and help to promote broad adoption and effective use of patient-facing APIs.

## Methods

### Recruitment

We created a convenience sample of health systems known to be early adopters of health technology, including health systems engaged in the Health Records on iPhone patient-facing API pilot (a list of health systems included in Apple’s press release) and others known to be the users of API technology (a list from authors’ professional networks), and then also selecting for regional variation by ensuring at least one health system per census region (Northeast, West, Midwest, and South). Although APIs can be used by patients, providers, payers, third-party apps, and others, we used “patient-facing APIs” to describe use cases where *patients* are the actors requesting EHI via the API, or directing third-party apps to access the information on their behalf. In business-to-business API use cases, health systems or other covered entities connect via API with other covered entities or business associates to exchange health data. Although the API itself might be technically identical in both use cases, the federal policies, data authorization workflows, and ultimately many of the specific use cases are different, which led us to examine patient-facing APIs separately.

### Interview Guides and Data Collection

We developed a semistructured interview guide that covered current patient-facing API efforts and capabilities, perceived barriers and risks, policy issues, organizational governance, and lessons learned (Multimedia Appendix 1). We reached out to the systems’ chief information officers (CIOs) by email, as these are the leaders who typically possess the greatest breadth of understanding of the topics and invited them or a designee with subject matter expertise on APIs to participate in the interview. We then shared the interview questions in advance and scheduled and conducted individual hour-long interviews. We included the first ten health systems that agreed to participate in interviews. We conducted interviews (by phone or videoconference) between September and December 2018. All interviews were recorded and transcribed. Our study was approved by the University of California, San Francisco, institutional review board (18-25416).

### Analysis

We performed a content analysis of interview transcripts using analytic matrices to extract relevant statements in three core topic categories related to our research domains: strategy and implementation, technical issues, and barriers and costs.

Specific topic categories within the strategy and implementation domain included (1) overall API strategy (both provider and patient-facing), (2) patient-facing API use cases, (3) status of implementation plan, (4) factors that shaped API strategy (organizational and policy), and (5) specific decisions and rationales for read and write APIs, white-listing or black-listing apps, publicity and marketing of APIs to patients and developers, and participation in Health Records on iPhone.

Specific topic categories within the technical issues domain included (1) level of effort required to implement patient-facing APIs, (2) level of EHR vendor engagement and support, (3) using APIs directly or with middleware, (4) staffing for API management, (5) one-time versus persistent access tokens, (6) ability to monitor the use of patient-facing APIs, and (7) technical lessons learned.

Specific topic categories within the barriers and costs domain included (1) barriers to and costs of implementing and using patient-facing APIs, (2) fees that the system assesses for the use of patient-facing APIs, and (3) fees that the EHR vendor assesses for the use of patient-facing APIs.

To minimize bias, we gave each respondent their own row in the matrices. We then identified themes within and across topic categories through discussion and consensus among the four authors. In some cases, we summarized the theme in a more concise, structured way and then reported the associated number of respondents with content supporting that theme. In other cases, we summarized the theme drawing more heavily on narrative. Under either approach, we supported the theme with example quotes. In the Results section below, we coded quotes with a unique but anonymous identifier per respondent, based on the respondent’s organizational role, for example, CIO1, CIO2, CTO1, and CTO2.

## Results

### Sample Characteristics

The health systems interviewed were located across the United States, with most in the Northeast or Midwest. Health system size was heterogeneous, with annual clinical revenues ranging from below US $10 billion to more than US $20 billion. Most health systems had an EHR from Epic Systems as their primary EHR, with a subset of these systems using additional EHRs in different locations because of affiliations, acquisitions, and regional consolidations. The leaders we interviewed covered a range of roles, including CIO, executive vice president, chief technology officer, senior medical director, and associate chief transformation officer (Table 1).

**Table 1:** Sample characteristics (n=10 organizations; 13 interviewees).

Characteristic		Value, n
**Geographic location**		
	Northeast	5
	Midwest	3
	West	1
	Southeast	1
**Size (discharges per year)**		
	<100,000	4
	100,000-200,000	3
	>200,000	3
**Total clinical revenue, US $**		
	<10 billion	3
	10-20 billion	3
	>20 billion	4
**Primary electronic health record vendor**		
	Epic	7
	Cerner	2
	Homegrown	1
**Role of interviewee**		
	Chief information officer	4
	Executive VP^a^	2
	CTO^b^	2
	Other (including executive director, senior medical director, chief digital officer, associate CTO, VP of information systems)	5

^a^VP: vice president.

^b^CTO: chief technology officer.

### Patient-Facing Application Programming Interface Strategies

Table 2 describes the API strategies of the organizations interviewed. Of the 10 organizations, eight had patient-facing APIs live and operational, while two were in the planning phase. Of the eight organizations with operational patient-facing APIs, five had delineated specific strategies (including dedicated technical support and explicit governance) for their use. The other three of eight organizations had activated patient-facing APIs but had a general API strategy for all users, not specific patient-facing API strategies or infrastructure. When we asked about API governance in more detail, seven organizations said that API decisions were made at the executive or board level, two organizations said that API decisions were made by technical teams (below the executive level), and one organization had created a dedicated API committee to make decisions about both patient-facing and business-to-business APIs.

We identified two broader themes related to organizational API strategies, which included factors and use cases shaping health systems’ strategies and encouraging their adoption and use of patient-facing APIs.

**Table 2:** Application programming interface strategies.

Type	Organizations, n	Description and example quotes
Operational patient-facing APIs^a^ with specific patient-facing API strategy	5	Defined strategy to support the technical implementation of patient-facing APIs and governance body with explicit oversight for patient-facing APIs Example quote: “It [API steering committee] originally started off as trying to launch the provider applications that connected to FHIR within our EHR and over time it has evolved to include some patient-facing use cases. The primary apps that it’s connected to is Apple Health.” [CIO1]
Operational patient-facing APIs without specific patient-facing API strategy	3	Patient-facing API strategy (with associated technology and governance) not distinct from overall API strategy Example quote: “We see this as part of a broader strategy of around getting the right data to the right person at the right time and don’t have a distinct strategy around that relative to use of APIs for other purposes.” [CIO2]
Planning for patient-facing APIs	2	Patient-facing APIs on the radar but specific implementation and governance plans are not yet developed. Example quote: “There is no conceptual reservation about doing this. We intend to do this. We will be opening up that consumer API and registering ourselves into the Apple and Google ecosystems as soon as we can.” [CTO1]

^a^API: application programming interface.

#### Theme 1: Diverse Factors Shape the Patient-Facing Application Programming Interface Strategy and Facilitate the Adoption and Use of Patient-Facing Application Programming Interfaces: Federal Policy and Regulations, Health Records on iPhone Availability, and Ethical Obligation

When asked about what shaped their strategy, three distinct and diverse factors were identified. The first factor was federal policy and regulations requiring patient-facing APIs, specifically the requirement that patients have access to and are able to use their health data through patient-facing APIs. As one interviewee explained:

I would say that CMS [Centers for Medicare & Medicaid Services] regulations is one of the top factors or rather what we feel like CMS regulation will become. That’s both in Meaningful Use and other kinds of regulations around interoperability []. I have to say that regulations do play a large influence.

CTO2

The second factor was Apple’s decision to include the option for patients to download their health record data to their Apple smartphone via Health Records on iPhone. A total of six organizations in our sample chose to partner with Apple to deploy this functionality, which required use of patient-facing APIs. The third factor was a value assessment or perceived ethical obligation to deploy functionality giving patients better access to their health data. As one interviewee stated:

We believe that patients should have their data in a format that is easy for them to share with who they want to share it with.

EVP1

#### Theme 2: Two Priority Use Cases Are Driving Patient-Facing Application Programming Interface Efforts

The first use case (cited by six organizations) includes use of patient-facing APIs to enable patients’ access to health information to facilitate a longitudinal health record combining information from multiple providers and integration of provider and payer data. Patient-facing APIs are uniquely capable of facilitating this use case (beyond an EHR-tethered patient portal) because they enable data integration from multiple sources. The second use case (cited by four organizations) includes the use of patient-facing APIs to enhance patients’ digital experience with the health system, including their ability to use convenience features such as telehealth, for example, transmitting health data to and from virtual visits and facilitating self-scheduling. For these systems, this use case aligns with consumers’ increasing expectations that health care, like other industries such as travel, food services, and financial services, provides data access and convenient self-service functionality that are widely available (primarily via mobile apps) [12,13].

When asked whether their organization’s primary use cases might change over the next 2 or 3 years, most interviewees thought that they would not. However, they did share specific ideas for how the functionality might evolve to deliver new value, in particular, by starting to enable better data-driven patient self-management. For example:

I think we’re going to see an explosion in more analytics and intelligence pushed out to the patient-facing APIs for self-triage management and escalation, as opposed to today where it comes across to the server side and we [health systems] have to manage it and triage it...I think it will be more proactive than reactive.

SMD1

Said another:

[In the future] if you check my data and you’re able to combine it with lifestyle data and other pieces of information that will add insights and value to my information beyond what my provider portal can do, then I am intrigued.

VP1

### Operational and Technical Features of Patient-Facing Application Programming Interfaces

Among the eight health systems with operational patient-facing APIs, we summarize key features of their operational and technical approaches and implementation decisions (Table 3). APIs can allow patients to access data (read) or provide data (write). All eight of these health systems had read APIs in operation. Although seven had at least some write capability, mostly focused on patients’ home device data, these implementations of write API functionality were variable, with some offering it only to a subset of invited patients rather than to all patients. There was also variation in whether patients could write data directly to the EHR or whether health system review and approval were required to authorize the data to be written into the EHR.

**Table 3:** Current status of patient-facing application programming interfaces (among eight organizations with operational application programming interfaces).

Category and status		Organizations, n	
**Types of APIs^a^ that are operational**			
	Read and write		4
	Read with limited access to write		3
	Read only		1
**App authorization**			
	White List: Health-System managed		4
	White List: EHR^b^ vendor managed		4
**Availability of API documentation**			
	From EHR vendor		4
	From health system		2
	No documentation available		2
**Patient communication about APIs**			
	Patient-facing APIs explained on health system website		2
**Fees^c^**			
	System charges or plans fees to patients for use		0
	EHR vendor charges system fees for patient-facing API use		0

^a^API: application programming interface.

^b^EHR: electronic health record.

^c^Although covered entities and their business associates under the Health Insurance Portability and Accountability Act may not charge patients a fee for electronic *access* to their health information through certified EHR technology—for example, to view, download, transmit, or access the health information through a patient-facing API—we still asked about actual practices in the field given the public reports and concerns about the fact and amount of fees being charged. Unlike this access, covered entities may charge patients a reasonable, cost-based fee for an electronic *copy* of their health information [14].

A second API implementation decision relates to how third-party apps are approved for API access. All eight health systems required prior approval of the app, or whitelisting, before an app could access the API. For four of these, the health system maintained the whitelist; for the other four, the EHR vendor maintained it. For third-party apps to connect to a patient-facing API, a developer requires documentation about the API configuration. A total of four health systems stated that this documentation was available to developers through their EHR vendor, with two health systems saying they would make it available to developers upon request and two health systems not having documentation available for developers. Of note, none of the health systems had publicly available API documentation for any developer to access. In all, two health systems noted that they were actively publicizing to their patient populations the availability of patient data via their patient-facing APIs, with one specifically noting its focus on how to utilize patient-facing API functionality to help “market to them the experience [rather than the underlying technology]” (SMD1).

In terms of more technical dimensions of patient-facing APIs, two health systems were relying on their EHR vendors to manage APIs, while six were using a middleware vendor for API management. API management refers to an array of capabilities, including API access control, API creation and design, API protection, business value reporting, and API traffic control, performance, and throttling [15]. Reliance on the EHR vendor rather than middleware appeared to be driven at least in part by EHR vendor capabilities, whether real or perceived, and possible differences between vendors. As one interviewee stated:

Some of the EHRs have made it easy, I would say [vendor 1] is one of them. Others are more difficult. I think its complex because it’s based on their internal resources, focus, structure, and capabilities.

CTO2

Another interviewee added:

You must have API middleware. [Otherwise], there is no way that you are going to be able to monitor the traffic flows or constrain traffic flows, or identify patterns. The EHR vendors are never going to figure this out.

CIO3

We also asked about health systems’ approaches to access tokens, which can be persistent (eg, log-in credentials not required each time the API attempts to read or write patient data) or one-time (ie, log-in required for each session the APIs are used). Overall, interviewees conveyed uncertainty about what types of tokens were in use, with two organizations explicitly not sure, two that thought they used persistent tokens, and three that thought they used one-time tokens.

Finally, when asked about fees associated with patient-facing APIs, none of the health systems were charging or planning to charge their patients a fee for use of patient-facing APIs—which was appropriate, given the regulatory prohibition against charging fees for patient access to their health information using patient-facing APIs in certified EHR technology. In addition, none of the health systems were paying specific fees to their EHR vendor for the use of patient-facing APIs.

### Perceived Barriers to the Current and Future Implementation and Use of Patient-Facing Application Programming Interfaces

We identified six items cited as barriers to implementation and use of patient-facing APIs (Table 4, Multimedia Appendix 2). The most common, cited by five health systems, was concerns about security or privacy introduced by enabling patient-facing APIs, such as unauthorized access or potential attacks by malicious actors. For example:

We worry a little bit that the spirit of the [regulation] is basically, well if any app comes knocking on your door, and the patient really wants to do this, then who are you to judge...[but] what if we can’t deliver it securely because the app is badly created or truly doing things like forking data off to a pharma company?

CIO3

Health systems felt unprepared to identify and differentiate the trustworthy from the untrustworthy app vendors, expressing additional uncertainty about whose responsibility this should become as the app ecosystem develops. Among our interviewees, some suggested an ongoing role for the EHR vendors, whereas others suggested instead a third-party rating consortium, with one suggesting “the creation of an expertise driven team to curate that portfolio would make the most sense” (CIO2). One element frequently tied to security concerns was a health system’s financial exposure, such as:

When something bad happens, they are not going to go after some little startup vendor. They are going to go after [health system]. So that’s why I think we have to be as clear as possible about what this decision means and where your information is going. You are taking this responsibility for this decision and we are not.

VP1

One health system interviewed had already taken a step toward trying to mitigate this, saying:

We do have a list of these recommendations and kind of a primer for patients for their digital health privacy...If we make the [health data] available, people should know how to unauthorize an application and know what to do if they suspect some app is doing something wrong with their data.

CIO1

Four health systems cited insufficient functionality in apps using patient-facing APIs as a barrier. Health systems believed that the current third-party app ecosystem did not yet offer a compelling alternative to EHR-tethered portals. For example:

First there need to be consumer apps that offer some added compelling value more than what the vendor mobile apps are doing.

ED1

Another interviewee said:

[We have] invested so much into the portal. [We are] concerned about the possibility of drawing some people away from it.

CTO1

A related barrier, cited by three health systems, was the lack of a perceived business value or financial return on investment, with one person saying:

[There is a] lack of external stimulus. It’s not a revenue driver. It’s not a cost reduction.

CTO1

Another said:

At some point if you have 100,000 different apps connecting with people, at some point someone is going to have to pay for those. And I don’t know exactly how that is going to get sorted out.

CIO1

Another barrier cited by three health systems was their EHR vendors’ wariness about data sharing. Said one executive:

Many vendors see data acquisition as a strategy for them so they don’t let you share.

EVP1

Said another:

[they think] patients will download malware that will take their patient data and do sinister things with it.

ED1

One system complained as follows:

[Y]ou [EHR vendor] need to open up your data so that we can do stuff in addition to what you are doing, and so that other people can do stuff to augment what you are doing.

CIO2

Finally, systems noted less evolved technology and standards in health care as a barrier. For example,

[T]he speed at which they [FHIR] are adding [data elements] is glacial. There are 4,000 data elements in [our EHR] and I think they’ve got maybe 150 done through FHIR [Fast Healthcare Interoperability Resources].

CIO2

Another interviewee added:

It’s sort of interesting that APIs really require that you build a sort of robustness and scalability that you’ve never really engineered for in the past because this all used to be done by a fairly finite population of doctors with fairly understandable behaviors, not thousands if not tens of thousands of people who...could update their medical records every six seconds...[FHIR] is so rapid in its evolution...and so suddenly you’re engineering a set of apps on shifting sands.

CIO3

There were concerns about available technical settings, such as:

We don’t have the ability to tell the patient for how long this application will have access to their data. That’s a pretty big factor.

VP1

Another specific concern related to less evolved technical capabilities was the fact that certain settings created in the EHR might not be transmitted across a patient-facing API. For example:

In the portal you can put in rules like don’t show cancer diagnosis until released by the doctor, but I hear in the HealthKit API all those filters are gone, so you may discover your cancer diagnosis on your phone before the doctor even knows.

CIO3

**Table 4:** Perceived barriers to the current/future implementation/use of patient-facing application programming interfaces.

Category	Organizations, n
Security or privacy concerns	5
Insufficient functionality available in third-party apps	4
Lack of perceived return-on-investment: costs vs business value	3
Vendor unwillingness to share data outside of their system	3
Technology not as evolved in health care vs other industries	3
Lack of application programming interface and semantic standards	2

## Discussion

### Principal Findings

In this study, we interviewed 10 leading health systems about their approach to patient-facing APIs to understand early experiences and identify insights for policy and practice. Our results suggest a reason for optimism about the prospects for patient-facing APIs and their impact on the US health care system. Building upon the progress made in the use of patient portals, patient-facing APIs further expand technical capabilities, leading us further toward the hopes inscribed nearly 2 decades ago in the Health Insurance Portability and Accountability Act Privacy Rule, which gave patients a right to access and use their full designated health record set.

The health systems we interviewed all planned to increase the use of patient-facing APIs, and many stated that this was “the right thing to do.” Two use cases emerged as the strategic driving forces for health systems: the ability for the patient to create an aggregated longitudinal health record and better digital patient engagement. However, we also found substantial heterogeneity in the strategy as well as operational and technical approaches. This suggests that there are not yet best practices to guide health systems on how to work with patient-facing APIs, nor clear business models driving a common strategic roadmap for their implementation. We also identified that there were commonly perceived barriers spanning a range of domains, suggesting that additional work is required, at both policy and practice levels, to ensure that patient-facing APIs successfully fulfill their intended purposes and use cases.

The implementation challenges and perceived risks we identified are leading organizations to act carefully and slowly around implementation and to avoid aggressively marketing the new functionality. Health systems commonly cited privacy and security concerns—a noteworthy perceived barrier as the regulations compel health systems to share electronic health data with patients via API to any third party and via any format requested by the patient. Health systems had associated concern about financial exposure. Specifically, even where patients have transmitted their data from the system to unaffiliated third-party apps and the system is not legally liable for the patient’s subsequent choices about disclosure and use, a patient might still choose to sue and may still garner a settlement given health systems’ “deep pockets.” Technical topics often brought out confusion about policy and implementation details, for example, with access tokens and whitelisting of apps, as well as sometimes opposing or divergent views, such as whether to use a middleware vendor rather than EHR vendor for API management. This is likely because there is not yet sufficient longitudinal experience with using patient-facing APIs for crisp, confident understanding of the technical nuances. The fact that some CIOs and other interviewees from large health systems with substantial technical experience are still on the learning curve about patient-facing APIs is an important finding from our work.

Health systems were also cautious about the market demand for the use of patient-facing APIs. Although they noted that patients expect more convenient tools for managing their care, the health systems we interviewed generally thought that their EHRs’ tethered portals still deliver greater patient convenience and value than the currently available third-party apps utilizing patient-facing APIs. From some health systems’ perspectives, a controlled, integrated system using an EHR-tethered portal seemed preferable to the potentially piecemeal, heterogeneous functionality of modular systems using patient-facing APIs. It is an open question, however, whether patients agree or would have an alternative assessment. For example, patients may want to choose their own appointment scheduling or cost-estimator app, or integrate their data across health systems, none of which is currently possible with a tethered portal.

On the basis of our findings, we believe additional actions are needed to spur health systems’ uptake and effective use of patient-facing APIs, primarily by tipping the balances toward increased upside and decreased downside for health systems. First, given the lack of clarity we found about some technical implementation best practices, we think ongoing dialog and sharing of experiences within the industry will be critical to bridge the heterogeneous technical approaches to implementation. For example, sharing experiences around the use of API middleware platforms vs embedded EHR vendor tools for API management would likely be valuable and could be facilitated by organizations such as the Health Services Platform Consortium, the College of Healthcare Information Management Executives, the Healthcare Information and Management Systems Society, the American Medical Informatics Association, or the Association of Medical Directors of Information Systems. Second, as we found frequent concerns about security, privacy, and health system liability; a broader public awareness campaign and tools to educate and aid the public in learning how to manage their health data may be needed. One specific strategy could feature increased commitment to use the Office of the National Coordinator for Health Information Technology’s (ONC) Model Privacy Notice to help consumers identify which apps may present higher privacy and security risk. Another strategy that might help convince health systems to expedite progress would be additional policy clarity and protections for health systems for data privacy breaches by apps chosen by the patient. Recent frequently asked questions published by the US Department of Health and Human Services are a good start in this direction [16]. A third strategy toward overcoming these concerns will be the emergence and development of expert consortia, making recommendations about the safety and efficacy of apps, to help health systems and patients understand which apps to use or avoid and thus aid adoption. It remains unclear whether this will be taken on by the ONC, EHR vendors, the Food and Drug Administration, hospital-specific “digital diagnostics and therapeutics committees” [17], multistakeholder coalitions, or some combination of all of these [18].

Finally, given that we found ongoing uncertainty about the relative value and cost of tethered EHR portals compared with third-party patient apps, we believe that there must be greater pressure on EHR vendors to expand the set of available APIs, even for functionality they believe might enable competitive products. This would require more rapid expansion of the set of data to be shared by standardized APIs, including the US Core Data for Interoperability (USCDI). Although ONC currently proposes to add three new data elements to the current set of 20, it could add other standardized data elements more rapidly, such as those that already exist as voluntary certified health information technology modules, for example, family history and social determinants of health, and focus on APIs that would facilitate patient-driven transactional interactions, such as appointment scheduling. Speeding these additions to the USCDI would more rapidly enable new apps to meet currently unmet needs, in turn driving patient interest and demand, leading to a virtuous cycle of increased health system prioritization of further implementation and use of patient-facing APIs.

### Limitations

Given that we studied a convenience sample of 10 early adopter health systems planning for or implementing patient-facing APIs, our results may be limited in their generalizability, and we may have failed to capture all important themes. Our sample was also skewed toward larger, urban health systems, which may fail to capture differences in experiences of smaller, more rural hospitals and health systems. We interviewed one person (or sometimes two) at each organization and left it up to the organization to select the most appropriate interviewees. As a result, respondents varied in their depth of knowledge about the broad range of topics covered, meaning certain quotes may reflect respondents’ perceptions.

### Comparison With Prior Work

Given the recent availability of patient-facing APIs, there is limited prior work on this topic. Four recent studies have assessed the uptake and use of APIs. One study examined the use of APIs across 12 large health systems and found relatively low, but steadily increasing, uptake [19]. Relevant to our findings, this study found very low use of APIs compared with levels of use of EHR-tethered patient portals. A second study, using the 2017 American Hospital Association data, found that 33% of hospitals reported that patients could access their EHI through EHR APIs [20]. A third study from Partners’ Healthcare system examined demographics of users and found that male patients and younger patients were more likely to use patient-facing APIs [21]. Finally, a study from the University of California, San Diego, surveyed the first 425 patients who had started using their personal health record API feature, with a very high percentage (75%-96%) of 132 survey respondents noting satisfaction with this new connectivity and the improved health informational benefits derived [22]. These findings complement our results in that initial results show slow uptake but significant enthusiasm for the potential benefits of patient-facing APIs.

### Conclusions

In summary, we found that diverse factors drive health systems’ efforts to pursue patient-facing APIs, including the benefits and opportunities for patient engagement and empowerment they bring to health care. However, we found substantial heterogeneity in the approaches by early adopter health systems, which are proceeding with caution and remain uncertain about multiple dimensions including the long-term business drivers. To ensure that national policy goals for interoperability and patient data access are met, there will need to be an ongoing understanding of the usage of and barriers to patient-facing APIs and increasing discussion and sharing of best practices, likely with targeted ongoing interventions to support and bolster their use.

## Appendix

Multimedia Appendix 1 Interview guide for “Early Insights from Health System Deployment of Patient-Facing APIs.”.

Multimedia Appendix 2 Results: barriers to implementation and use.

## Abbreviations

API: application programming interface
CIO: chief information officer
EHI: electronic health information
EHR: electronic health record
ONC: Office of the National Coordinator for Health Information Technology
USCDI: US Core Data for Interoperability
