# Supplementary material for: Deploying Patient-Facing Application Programming Interfaces: Thematic Analysis of Health System Experiences
Source: J Med Internet Res. 2020 Apr 3;22(4):e16813. doi: 10.2196/16813 (PMC7165308; doi:10.2196/16813)
Supplement: Multimedia Appendix 1 [file jmir_v22i4e16813_app1.docx]

**Appendix: Interview Guide for “An Early Look at Patient-Facing APIs”**

**Section 1: Background, context, and governance**

1. What is the size and scope of services provided by your organization?
2. What is your role in your organization?
3. Who are the primary vendors in your current clinical IT environment?
4. What terminology is used at your organization to describe patient-facing APIs in comparison to “B2B” APIs?
5. Who in your organization is responsible for:
   - strategic oversight of patient-facing APIs?
   - use case development for patient-facing APIs?
   - build, maintenance, and support for patient-facing APIs?

**Section 2: Patient-facing FHIR API strategy, use cases, and status**

1. Does your organization have a defined strategy for implementation and use of patient-facing APIs?
   - What does your organization see as the current primary and secondary use cases and purposes of these APIs?
     - Do you expect these primary and secondary use cases to change at all in the next 2-3 years?
   - If you have multiple EHRs within your organization, are you using or planning to use patient-facing APIs to create a single patient portal experience for patients?
   - Are you considering using patient-facing APIs as a way to consume patient-generated health data?
   - Are you considering using patient-facing APIs as a method for release of information (e.g., medical records requests)?
2. Where are you currently in this implementation plan?
3. Has your organization “turned on” any patient-facing APIs yet?
   - If yes, which ones? (eg Read, Write?)
   - If no, what is the current status of the access/availability of these APIs?
4. What factors shaped your organization’s API strategy in terms of use cases, timing, etc?
5. What sources of information did you use to inform your API strategy and implementation plan? Which were the most helpful sources or resources?
6. Please describe the role that your EHR vendor plays in your patient-facing API strategy and implementation, in terms of:
   - Content and breadth of data (i.e., What data can be shared through an API? If, for example, you wanted to release clinical notes through your API, how, if at all, would you need to engage your vendor?)
   - Who controls the “white listing” or “black listing” of third-party applications?
   - What are the associated costs?
7. If you are white-listing or black-listing applications, how are you making those decisions?
8. Is your organization publicizing or actively marketing the availability of these APIs? To patients? To partners? To developers?
9. Are your patient-facing APIs open, with complete documentation available to the public? Is anything hidden?
   - Has your organization signed on to connect to Apple Health? What led you to decide to sign up with (or decline to sign up with) Apple?
   - Who was involved in the decision-making?
   - What costs have you borne in working with Apple to implement the APIs?
10. Are there any data elements or use cases for patient-facing APIs over the next two years that you think are valuable, but you won’t be able to pursue? If so, what are the impediments?

**Section 3: Technical**

1. Who is responsible for the implementation of patient-facing APIs for your organization?
2. What type of staff and skills are necessary for the implementation and set-up? Do you have access to these staff and skills?
3. What level of effort and complexity have you found is required to implement these patient-facing APIs? Or, what effort are you estimating? Is the more/less than expected?
4. If not already discussed, how did your EHR vendor facilitate this process? Where could you have used additional help beyond what the vendor provided?
5. Are you using patient-facing APIs “out of the box” from your EHR vendor, or are you using any additional vendors, such as middleware, API managers, or ESB vendors?
6. In a broader sense, who do you feel is best positioned to manage APIs – organizations like yours, EHR vendors, large companies with substantial API experience (e.g., Microsoft, Amazon, Google, Mulesoft), or others? How do you anticipate that this will evolve over time?
7. Have you had any technical “lessons learned” from implementing patient-facing APIs?
8. What is your approach to one-time versus persistent tokens? What are your motivations for these decisions?
9. Are you able to monitor use of your patient-facing APIs?
   - If so, what volume of use are they getting?
   - Which types of third party applications are most-frequently being used to connect to the patient-facing APIs?
10. In your experience, do some kinds of patient-facing applications work better with APIs than others? Which and why?
11. What have your customers/stakeholders told you, if anything, about your patient-facing APIs?

**Section 4: Perceived barriers and risks**

1. What are the largest barriers to your organization’s current and future development or use of patient-facing APIs?
2. What are the barriers and risks you are most concerned about?
   - Within your organization, what governance, technical, or other strategies would mitigate these barriers and risks?
   - Are there public or private-sector efforts that could help mitigate these barriers and risks?
3. What costs are you budgeting for patient-facing API efforts and what general expenses do you anticipate?
4. Is your organization assessing fees to anybody for use of patient-facing APIs? To whom? How are the fees determined?
5. Is your EHR vendor assessing fees to anybody for use of patient-facing APIs? To whom? How are the fees determined?

**Section 5: Policy**

1. What policy forces are shaping your patient-facing API strategy?
   - What is the impact of 2015 Edition of certified health IT? What is the impact of CMS delay from 2018 to 2019? What expectation of use in 2018 because of MIPS 10% bonus? What expectations of use in 2019?
   - 21^st^ Century Cures requires APIs that allow health information to be accessed, exchanged and used “without special effort”:  What does “without special effort” mean to you, how do your APIs help implement/ensure that?
   - What about new payment models (e.g., ACOs), VA API efforts, others?
