# Supplementary figures and images for: Deploying Patient-Facing Application Programming Interfaces: Thematic Analysis of Health System Experiences
Source: J Med Internet Res. 2020 Apr 3;22(4):e16813. doi: 10.2196/16813 (PMC7165308; doi:10.2196/16813)

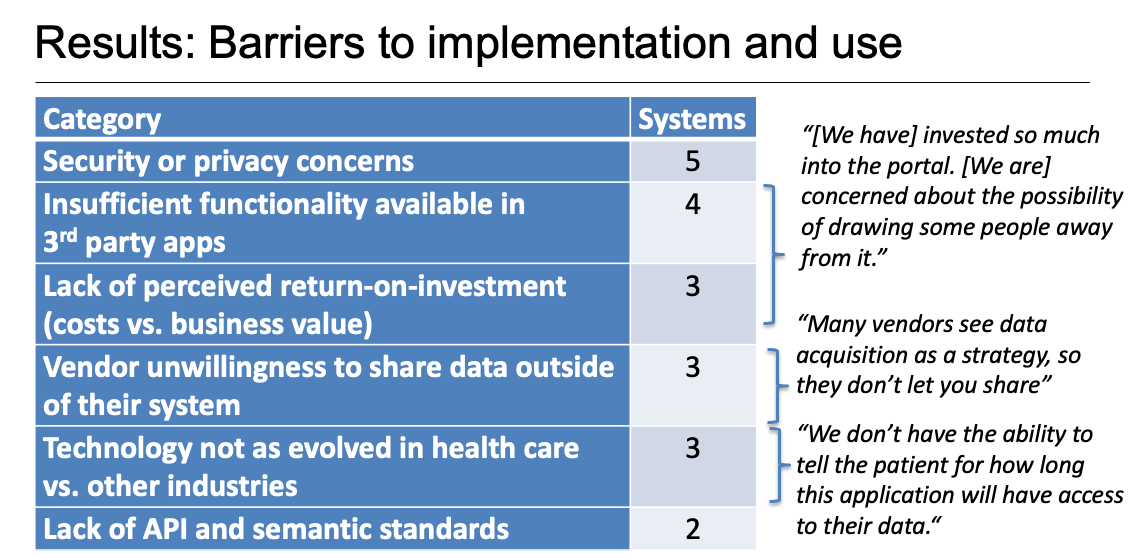

Supplement: Multimedia Appendix 2 [file jmir_v22i4e16813_app2.png]
